# Aberrant histone acetylation and dysregulated synaptic plasticity in cognitive impairment induced by a high-methionine diet

**DOI:** 10.4103/NRR.NRR-D-25-00069

**Published:** 2025-09-03

**Authors:** Jianting Li, Yuan Fu, Xiaolong Gu, Qi Xie, Zengli Liu, Zhihua Cao, Lu Li, Jiaxin Ren, Yang Li, Hailan Yang, Zhiwei Peng, Zhizhen Liu, Jun Xie

**Affiliations:** 1 Department of Biochemistry and Molecular Biology, College of Basic Medicine, Shanxi Key Laboratory of Birth Defect and Cell Regeneration, MOE Key Laboratory of Coal Environmental Pathogenicity and Prevention, Shanxi Medical University, Taiyuan, Shanxi Province, China; 2 Department of Nephrology, First Clinical College of Shanxi Medical University, Taiyuan, Shanxi Province, China; 3 Department of Obstetrics, First Clinical College of Shanxi Medical University, Taiyuan, Shanxi Province, China

**Keywords:** cognitive impairment, high-methionine diets, histone H3 lysine 27 acetylation, homocysteine, nerve regeneration, synaptic long-term potentiation

## Abstract

Cognitive impairment is a complex neurodegenerative disorder, and increased homocysteine levels are recognized as a major risk factor for this condition. Epigenetic modifications, particularly histone acetylation, have been implicated in the progression of cognitive impairment; however, the mechanisms underlying hyperhomocysteinemia-induced cognitive impairment remain unclear. In this study, we developed an hyperhomocysteinemia-induced cognitive impairment model by feeding mice a high-methionine diet and conducted behavioral and molecular analyses to elucidate the mechanisms involved in cognitive impairment. Behavioral experiments revealed significant cognitive deficits and neuroinflammation accompanied by a marked decrease in histone H3 lysine 27 acetylation in the hippocampus and cortex. Furthermore, metabolomic profiling and chromatin immunoprecipitation sequencing demonstrated substantial shifts in the levels of homocysteine metabolites and identified histone H3 lysine 27 acetylation-targeted genes involved in synaptic long-term potentiation, including *Gria1*, *Gria3*, *Grin2a*, *Grin2b*, *Slc1a1*, *Slc24a2*, *Ptk2b*, and *Src*. RNA sequencing confirmed that hyperhomocysteinemia induced neurodegeneration. *In vitro* experiments confirmed that decreased histone H3 lysine 27 acetylation downregulates the expression of these target genes in homocysteine-treated HT-22 cells, thereby impairing synaptic plasticity. Collectively, these findings suggest that aberrant expression of long-term potentiation-related genes regulated by histone H3 lysine 27 acetylation is a key driver of hyperhomocysteinemia-induced cognitive impairment. Targeting histone H3 lysine 27 acetylation-mediated epigenetic dysregulation may be a promising therapeutic strategy, offering potential avenues for intervention in individuals with cognitive impairment and neurodegenerative disorders.

## Graphical abstract

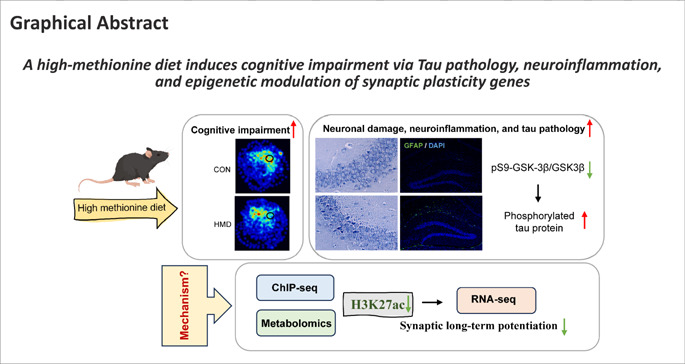

## Introduction

Neurodegenerative diseases (NDDs) constitute a diverse group of neurological disorders that impact millions of individuals worldwide and are characterized by progressive degeneration or loss of neurons within the central and peripheral nervous system (Wilson et al., 2023). NDDs, which are associated with considerable morbidity and cognitive impairment, have an estimated prevalence of 9.33% worldwide (Wimo et al., 2017). Alzheimer’s disease (AD) (Long and Holtzman, 2019; Knopman et al., 2021), Parkinson’s disease (PD) (Poewe et al., 2017; Przedborski, 2017), Huntington’s disease (HD) (Bates et al., 2015; Jimenez-Sanchez et al., 2017), and amyotrophic lateral sclerosis (ALS) (Kiernan et al., 2011; Rohrer et al., 2015) are the most common NDDs. Neural network collapse and the loss of neurons, which cannot be regenerated owing to their terminally differentiated state, disrupt core communication pathways, leading to deficits in memory, cognition, behavior, sensory processing, and/or motor function (Erkkinen et al., 2018). Thus, identifying the etiology and underlying molecular mechanisms of cognitive impairment in NDDs is important for developing effective therapies to improve patients’ quality of life.

High levels of homocysteine (Hcy), a physiological amino acid generated during protein metabolism, cause a pathological condition known as hyperhomocysteinemia (hHcy), which was first reported as a factor contributing to cardiovascular disease (Ganguly and Alam, 2015; Gospodarczyk et al., 2022). Recent evidence has demonstrated that hHcy is epidemiologically and clinically associated with various pathological conditions and is a risk factor for NDDs (Schalinske and Smazal, 2012; Cordaro et al., 2021). Methionine (Met) is an essential amino acid that is involved in Hcy production via the methionine cycle and is a key methyl donor in cellular metabolism (Froese et al., 2019). Chronic consumption of a high-methionine diet (HMD) induces a state of hHcy, which may promote amyloid-β (Aβ) overproduction, Tau protein hyperphosphorylation, neuronal loss, inflammation, and oxidative stress, leading to cognitive impairment (Grinan-Ferre et al., 2018; Qureshi et al., 2019; Wang et al., 2022). Hcy is generally thought to induce cell damage via oxidative stress (Kolling et al., 2011; Scherer et al., 2011). Specifically, Hcy-induced oxidative stress has been linked to energy metabolism changes in NDDs (Zhou et al., 2023). Hcy is a physiological metabolite of the methionine cycle, which generates S-adenosylmethionine, the most important methyl donor for DNA methylation and epigenetic regulation (Froese et al., 2019). However, it is unclear whether Hcy regulates histone modification in the context of cognitive impairment.

Posttranslational modifications (PTMs) of protein lysine residues (e.g., acetylation, succinylation, lactylation, and malonylation) play important roles in regulating cellular functions in both physiological and pathological states (Wang and Lin, 2021; Fu et al., 2022). Acetylation is a PTM that is involved in the regulation of both histone and non-histone proteins. Acetylation is crucial for maintaining neuronal plasticity and plays an essential role in memory formation and learning (Kabir et al., 2022). Acetylation homeostasis is regulated by the coordinated activities of histone acetyltransferases (HATs) and histone deacetylases (HDACs). Dysregulation of these tightly controlled processes has been implicated in several NDDs, including AD, PD, HD, and ALS (Khan et al., 2021; Kabir et al., 2022; Lin et al., 2023). Chai et al. (2024) reported that, in hHcy rats, levels of the permissive histone marker trimethyl histone H3 lysine 4 and its methyltransferase lysine methyltransferase 2B were significantly increased, impairing synaptic plasticity and cognitive function, which highlights the role of histone acetylation in Hcy-related cognitive disorders. In addition, histone H3 lysine 27 acetylation (H3K27ac) and H3K9ac disrupt feedback loops that regulate the expression of transcription- and chromatin-related genes in AD, suggesting potential epigenetic strategies for early-stage AD treatment (Nativio et al., 2020). In HD models, transcriptional and histone acetylation–related alterations highlight key cis-regulatory elements factors affecting disease pathology in the early stages (Arancibia-Opazo et al., 2023). These studies suggest important roles for histone acetylation and its associated enzymes in NDDs. However, the specific mechanism by which histone acetylation modifies Hcy-induced cognitive impairment remains unclear.

The aim of the present study was to identify novel epigenetic regulatory targets or strategies for the diagnosis and treatment of cognitive impairment in Hcy-related NDDs. We explored relationships among Hcy metabolism, H3K27ac modification, and cognitive impairment in HMD mice, integrating and connecting these factors via unique approaches such as chromatin immunoprecipitation sequencing (ChIP-seq), metabolomics, and behavioral experiments. Our findings improve understanding of the etiology of cognitive impairment, providing new insight and facilitating the exploration of potential diagnostic and therapeutic targets for reversing cognitive impairment in patients with NDD.

## Methods

### Animals

Epidemiological studies have shown that two-thirds of patients with dementia are female, and the lifetime risk of developing dementia for women is approximately twice that of men (Scheltens et al., 2021). Therefore, this study used specific pathogen-free C57BL/6 adult female mice (8-week-old) weighing 18–20 g, purchased from the Experimental Animal Center of Shanxi Medical University (license No. SCXK (Jin) 2019-0004). All animal experiments were conducted in a barrier environment at the Experimental Animal Center of Shanxi Medical University. Every 5 mice were housed dour per cage at a stable temperature (24 ± 2°C), with 12-hour/12-hour light/dark time alternation and a constant humidity of 40%–70%. The mice were randomly divided into two groups: the control group fed a standard diet (main components include 23.07% protein, 11.85% fat, and 65.08% carbohydrates, Beijing Keao Xieli Feed Co., Ltd., Beijing, China) (CON, *n* = 30); and the cognitive impairment model group fed a high-methionine diet (main components include 2% methionine, Beijing Keao Xieli Feed Co., Ltd.) (HMD, *n* = 30) for 8 weeks. The mice were allowed *ad libitum* access to food and water. The research protocol was approved by the Experimental Animal Ethics Committee of Shanxi Medical University (approval No. SYDL2025003) on January 10, 2025, and conducted in accordance with the National Institutes of Health Guide for the Care and Use of Laboratory Animals (8^th^ ed., National Research Council, 2011).

### Mouse behavioral tests

All mice were acclimatized for 1 day prior to performing the experiments. The behavioral test apparatus was sanitized with 75% ethanol and air-dried before use. All behavioral data were analyzed statistically using an EthoVision XT 15 system (Noldus Information Technology, Wageningen, the Netherlands).

### Nesting ability test

The nesting test assessed autonomous behavior by placing each mouse in a cage containing corn cobs and a piece of cotton (5 × 5 × 1 cm^3^ thick) as nesting material (Deacon, 2006). Nesting behavior was evaluated after 24 hours according to a definitive five-point nest-rating scale. The scoring was performed by three independent observers who were unaware of the group assignments.

### Open field test

The open field test was used to assess the motor ability of mice by placing each mouse in the central area of a cubic test box (40 × 40 × 40 cm^3^). Each mouse’s free movement was recorded for 5 minutes using a video camera (Zhu et al., 2018).

### T-maze test

A T-maze was used to evaluate the short-term spatial learning and memory abilities of mice by testing their spontaneous activity and voluntary choices (Deacon and Rawlins, 2006). The T-maze consists of three arms (40 × 8 × 15 cm^3^). At the beginning of the experiment, each mouse was placed in the starting box at the end of the starting arm.

Spontaneous alternation behavior test: mice explored the T-maze freely for 8 minutes while their activities were recorded. Alternation accuracy was calculated using the following formula: alternation accuracy = number of correct arm entries/(total number of arm entries – 2) × 100%. Novel arm exploration experiment: during training, the novel arm was blocked, and mice moved freely in the maze for 10 minutes. After 1 hour, the baffle was removed, and mice were allowed to explore the maze for 5 minutes. The number of entries into the novel arm was recorded.

### Novel object recognition test

The novel object recognition test evaluates visual recognition learning and memory in mice, based on their tendency to explore new objects (Xiao et al., 2023). The experiment was conducted in a cubic box (40 × 40 × 40 cm^3^) over three phases. During the adaptation phase, on day 1, mice were acclimated to the empty box for 10 minutes. For the familiarization phase on day 2, mice freely explored the box with two identical objects for 10 minutes. For the test phase on day 3, one object was replaced with a novel object, and mice were allowed to explore for 10 minutes. Long-term memory was assessed 24 hours later. Mice spending less than 10 seconds exploring both objects on day 3 were excluded. The novel object recognition rate was calculated as follows: (number of novel object explorations/total explorations) × 100%. The recognition index was calculated as: (time spent on the novel object/total exploration time) × 100%.

### Morris water maze test

The Morris water maze test was used to assess the long-term spatial learning and memory ability of mice (Pi et al., 2021). The experiment was conducted in a white circular pool (120 cm in diameter) filled with water at 22 ± 2°C, with a depth of approximately 25 cm. A circular platform, submerged 1 cm below the water surface, was placed in the northeast quadrant. Data for each mouse were automatically recorded using a camera system.

The experiment consisted of a 4-day training period followed by a test on the fifth day. During training, mice were placed in the water in different quadrants, facing the wall, for a 60-second trial. If a mouse located the platform within 60 seconds and remained on it for 3 seconds, the trial ended, and the escape latency (time to find the platform) was recorded. Mice failing to locate the platform were guided to it and left there for 20 seconds, and their escape latency was recorded as 60 seconds. The daily performance was averaged across the four quadrants. On the fifth day, the platform was removed, and mice were tested for latency to the first platform crossing and the number of crossings within 60 seconds.

After the behavioral tests were complete, the mice were fasted for 14–16 hours and anesthetized by intraperitoneal injection of sodium pentobarbital (50 mg/kg), and blood samples were collected from the retro-orbital plexus. Brain, heart, liver, and kidney tissues were harvested, and the organ index was calculated as follows: organ index = organ weight (mg)/body weight (g) × 100%. A portion of the brain was fixed in 4% paraformaldehyde, while the cortex and hippocampus were isolated from the remaining brain tissue and stored at –80°C.

### Cell culture and homocysteine treatment

A mouse hippocampal neuron cell line (HT-22, RRID: CVCL0321) was purchased from Jennio Biologicol Technology (Guangzhou, China). The cells were cultured in Dulbecco’s Modified Eagle’s Medium (DMEM, Gibco, Grand Island, NY, USA) containing 10% fetal bovine serum, 1% streptomycin–penicillin solution, and 1% non-essential amino acids (complete medium). The cells were subjected to the following interventions: CON group (normal culture for 24 hours), HCY group (2 mM Hcy treatment for 24 hours, H4628, Sigma-Aldrich, St. Louis, MO, USA), and HTL group (2 mM homocysteine thiolactone [HTL] treatment for 24 hours, H6503, Sigma-Aldrich).

### Cell Counting Kit-8 and scratch assay

HT-22 cell proliferation was assessed using a Cell Counting Kit-8 (CCK-8; BA00208, Bioss, Beijing, China). Cells were seeded into 96-well plates (5 × 10^3^ cells/well) and treated with 100 μM, 500 μM, 1 mM, or 2 mM HTL or Hcy for 24 hours once they reached 70% confluence. A mixture of 10 μL CCK-8 solution and 90 μL complete medium was added to each well. After 1 hour of incubation, absorbance was measured at 450 nm using a microplate reader (Molecular Devices, Sunnyvale, CA, USA).

HT-22 cell migration was evaluated by scratch assay. Five evenly spaced lines were marked on the back of six-well plates. Cells (3 × 10^5^ cells/well) were seeded into the wells and treated for 24 hours after reaching 70% confluence. A vertical scratch was made over each mark on the plate with a 200-μL pipette tip, followed by washing and culturing in serum-free complete medium. Images were captured at 0, 12, 24, 36, and 48 hours using an inverted microscope (Nikon, Tokyo, Japan) and analyzed using ImageJ software (version 1.8.0.112; National Institutes of Health, Bethesda, MD, USA). The migration rate was calculated as follows: cell migration rate (%) = (scratch area at 0 hours – scratch area at each time point)/scratch area at 0 hours × 100.

### Crystal violet staining

HT-22 cells were fixed with 4% paraformaldehyde for 10 minutes, washed with the phosphate-buffered saline (PBS, Servicebio, Wuhan, Hubei, China), and then stained with crystal violet staining solution (C0121, Beyotime, Shanghai, China) for 10 minutes. Images were captured using an inverted microscope (Nikon).

### Apoptosis detection

The cell apoptosis rate was determined using Annexin V-EGFP/PI Apoptosis Detection Kit (KTA0005, Abbkine, Wuhan, Hubei, China). HT-22 cells were treated as described above for 24 hours and digested with EDTA-free trypsin. After washing with PBS, the cells were resuspended in 100 μL Annexin V binding buffer, stained with Annexin V-FITC and PI, and analyzed by flow cytometry (BD FACSCelesta, Franklin Lake, NJ, USA). The apoptosis rate was calculated as follows: cell apoptosis rate = percentage of lower right quadrant (early apoptotic cells) + percentage of upper right quadrant (late apoptotic cells).

### Tissue preparation

After fixation, the brain tissue was embedded in paraffin and sliced into 5-μm-thick sections according to routine procedures. The sections were baked at 65°C for 2 hours, followed by immersion in xylene and a graded ethanol series, followed by rinsing with PBS.

### Enzyme-linked immunosorbent assay for homocysteine and lipid metabolism indicators

The Hcy concentration in serum, brain tissue, and cells was determined using a Mouse Hcy ELISA Kit (LV30834, Animalunion Biotechnology, Shanghai, China), and serum triglyceride, total cholesterol, high-density lipoprotein, and low-density lipoprotein levels were determined using kits according to the manufacturer’s instructions (A111-1-1, A110-1-1, A113-1-1, and A112-1-1; Njjcbio, Nanjing, Jiangsu, China).

### Hematoxylin–eosin staining and Nissl staining

Hematoxylin–eosin (HE) staining (G1003, Servicebio) and Nissl staining (G1036, Servicebio) were used to observe pathological changes in the cortex and hippocampus with an optical microscope (Olympus, Tokyo, Japan).

### Immunostaining

Antigen retrieval (acidic or alkaline) for brain tissues was performed five times (3 min each) based on the primary antibody requirements. After washing with PBS, sections were blocked with 5% goat serum containing 1% Triton-X-100 at room temperature for 1 hour. Then, the sections were incubated overnight at 4°C with rabbit an anti-H3K27ac (1:200, CST, Boston, MA, USA, Cat# D5E4) or rabbit anti-glial fibrillary acidic protein (GFAP; 1:200, Proteintech, Wuhan, Hubei, China, Cat# 16825-1-AP, RRID: AB_2109646) antibody. The next day, the sections were treated with Alexa Fluor® 488-labeled goat anti-rabbit IgG (1:200, ZSGB-BIO, Beijing, China, Cat# ZF-0511, RRID: AB_2864279) at room temperature in the dark for 1 hour. Coverslips were mounted on the slides using Antifade Mounting Medium containing 4′,6-diamidino-2-phenylindole (DAPI; P0131-5ml, Beyotime). Fluorescence signals were captured using a laser scanning confocal microscope (Leica TCS SP8) and analyzed with ImageJ.

For immunohistochemistry, the sections were incubated in 3% H_2_O_2_ at room temperature in the dark for 15 minutes, washed with PBS, and blocked with 5% goat serum for 1 hour. They were then incubated overnight at 4°C with a rabbit anti-pT231-Tau (1:200, Beyotime, Cat# AF1951) or rabbit anti-glycogen synthase kinase-3β (GSK3β; 1:200, CST, Cat# D5C5Z) antibody. The next day, the sections were treated with horseradish peroxidase–conjugated goat anti-rabbit IgG (1:500, Bioss, Cat# bs-80295G-horseradish peroxidase) for 1 hour at room temperature. Tissue staining was performed using a 3,3′-diaminobenzidine color development kit (ZLI-9019, ZSGB-BIO), and coverslips were mounted on the slides using neutral resin. Images were captured using an optical microscope (Olympus) and analyzed with ImageJ.

### Chromatin immunoprecipitation, chromatin immunoprecipitation sequencing, and chromatin immunoprecipitation-quantitative polymerase chain reaction

ChIP experiments were performed using a SimpleChIP® Plus Enzymatic Chromatin IP Kit (Magnetic Beads) (CST, Cat# 9005) according to the manufacturer’s instructions. The tissue samples were treated with formaldehyde to crosslink chromatin, and the chromatin was fragmented by partial digestion with micrococcal nuclease to obtain fragments containing one to five nucleosomes. ChIP was performed using ChIP-validated rabbit anti-H3K27ac (1:100, CST, Cat# D5E4) and ChIP-Grade Protein G Magnetic Beads. After reversal of the protein–DNA cross-links, the DNA was purified using DNA purification spin columns (CST, Cat# 14209).

H3K27ac ChIP-seq data consisting of raw fastq files were obtained from Gene Expression Omnibus (GEO) under accession number GSE280827. ChIP-seq libraries were prepared by Novogene Corporation (Beijing, China) using the KAPA HTP Library Preparation Kit complemented with NEXTflex DNA Barcodes from Bio Scientific (Austin, TX, USA). Subsequently, paired-end sample sequencing was performed on an Illumina platform (Illumina, San Diego, CA, USA). Library quality was assessed using an Agilent Bioanalyzer 2100 system (Santa Clara, CA, USA). The reference genome index was built using BWA (v 0.7.12; https://bio-bwa.sourceforge.net/), and clean reads were aligned to the reference genome using BWA mem (v 0.7.12) to identify DNA segments that interacted with H3K27ac across the entire genome.

Chromatin immunoprecipitation-quantitative polymerase chain reaction (ChIP-qPCR) was performed using purified DNA and 2× M5 HiPer Realtime PCR Super mix with Low Rox (SYBRgreen, with anti-Taq) (MF797-05, Mei5bio, Beijing, China). Two percent of the chromatin was removed for use as the input control, and ChIP signals were calculated as percentages of the input. The primer sequences used for ChIP-qPCR are listed in **Additional Table 1**.

**Additional Table 1:** Primers used for chromatin immunoprecipitation-quantitative polymerase chain reaction

Name	Sequence (5' to 3')
Mouse-Gria1	Forward: TAGCCTCTTGGGGTCTCTCC Reverse: AGCTCACGCACATGTACACA
Mouse-Gria3	Forward: GACTGGGTGAGTTGGGAGTG Reverse: CACCTTCCCTGCAAGAGGAG
Mouse- Grin2a	Forward: TCACCCACCACCAGGCTATA Reverse: CCAGGAGCAGGCAAGTGTAA
Mouse-Grin2b	Forward: GCTTTTTGCCCTCACCCTTG Reverse: AACCACATCCTTAGCCCTGC
Mouse-Slc1a1	Forward: TCTGTGGCTAGATCTGGGCT Reverse: CCCACAGTGCTTTTTGGAGC
Mouse-Slc24a2	Forward: GGGAGAGGAGAAGGCTACCA Reverse: GGTAGCCAGTCACAGCAGTT
Mouse-Ptk2b	Forward: AGGAGAAATCTGTGGCCAGC Reverse: AGGGTCCTGGATCCTGAGAC
Mouse-Src	Forward: CACCCCAATGAGTGTACGCT Reverse: TCCTGCTATGTACCCAGCCT

### Metabolomics analysis

Metabolomics was performed based on ultra-performance liquid chromatography coupled with high-resolution mass spectrometry (LC-MS/MS) technology to maximize the amount of total metabolite information obtained from the mouse hippocampus samples. The raw mass spectrometry data were converted into mzXML format using ProteoWizard (https://proteowizard.sourceforge.io/), and peak extraction, alignment, and retention time correction were performed using XCMS software (Novogene, Tianjin, China). The total peak area extracted from the sample was corrected and filtered, and the metabolomics data were analyzed using the NovoMetDB-UM-V3.0 database, a proprietary metabolome library developed by Novogene (Beijing, China). Subsequently, multivariate statistical analysis of the metabolites was performed to identify differences in metabolic patterns between the hippocampi of the two groups of mice and the biological significance of the metabolites.

### RNA extraction and quantitative reverse transcription-polymerase chain reaction

Total RNA was extracted from hippocampal tissue, cortical tissue, and cells using TRNzol (TIANGEN, Beijing, China). Total RNA was then reverse transcribed using 5× All-In-One RT MasterMix (ABS-G490, ABM, Vancouver, Canada), and quantitative reverse transcription-polymerase chain reaction (qRT-PCR) was performed using 2× M5 HiPer Realtime PCR Super Mix with Low Rox (SYBRgreen, with anti-Taq) (MF797-05, Mei5bio) on a QuantStudio 3 real-time PCR instrument. The relative mRNA expression level was calculated using the 2^–ΔΔCt^ method, with glyceraldehyde 3-phosphate dehydrogenase (GAPDH) as the reference gene. The primer sequences used for qRT-PCR are listed in **Additional Table 2**.

**Additional Table 2:** Primers used for quantitative reverse transcription-polymerase chain reaction

Name	Sequence (5 'to 3')
GAPDH	Forward: TGACCTCAACTACATGGTCTACA Reverse: CTTCCCATTCTCGGCCTTG
Gria1	Forward: GTCCGCCCTGAGAAATCCAG Reverse: CTCGCCCTTGTCGTACCAC
Gria3	Forward: CTCCAGAGGGGTGTATGCTAT Reverse: GTGACGAAAGATGTATGCAGGG
Grin2a	Forward: ACGTGACAGAACGCGAACTT Reverse: TCAGTGCGGTTCATCAATAACG
Grin2b	Forward: GCCATGAACGAGACTGACCC Reverse: GCTTCCTGGTCCGTGTCATC
Slc1a1	Forward: CTTCCTACGGAATCACTGGCT Reverse: CGATCAGCGGCAAAATGACC
Slc24a2	Forward: GACAAGATTCGAGATTACACCCC TGCAGAATGATGGCACCTTTC
Ptk2b	Forward: TGAGCCCTTGAGCCGTGTA Reverse: AGCTTGAAGTTCTTCCCTGGG
Src	Forward: GAACCCGAGAGGGACCTTC Reverse: GAGGCAGTAGGCACCTTTTGT
CBP	Forward: GGCTTCTCCGCGAATGACAA Reverse: GTTTGGACGCAGCATCTGGA
P300	Forward: TTCAGCCAAGCGGCCTAAA Reverse: CGCCACCATTGGTTAGTCCC
HDAC1	Forward: AGTCTGTTACTACTACGACGGG Reverse: TGAGCAGCAAATTGTGAGTCAT
HDAC2	Forward: GGAGGAGGCTACACAATCCG Reverse: TCTGGAGTGTTCTGGTTTGTCA
HDAC3	Forward: GCCAAGACCGTGGCGTATT Reverse: GTCCAGCTCCATAGTGGAAGT

CBP: CREB-binding protein; GAPDH: glyceraldehyde 3-phosphate dehydrogenase; HDAC: histone deacetylases.

### RNA sequencing

Total RNA was extracted, quantified (NanoDrop 2000; Thermo Fisher Scientific, Waltham, MA, USA), and ribosomal RNA was depleted using the Epicenter Ribo-Zero Gold Kit (Illumina, San Diego, CA, USA). Sequencing libraries were prepared by Novogene Corporation (Beijing, China) using the TruSeq Stranded mRNA Library Prep Kit (Illumina), followed by PCR amplification with indexed primers. Library quality was assessed using an Agilent Bioanalyzer 2100 system (Agilent Technologies, Santa Clara, CA, USA), and paired-end sequencing (150 bp) was performed on an Illumina NovaSeq 6000 platform (Illumina). Clean reads were aligned to the reference genome ([Species/Version]) using HISAT2 (v2.2.1; https://daehwankimlab.github.io/hisat2/), and gene expression quantification was performed with featureCounts (v2.0.1; https://subread.sourceforge.net/). Differentially expressed genes (DEGs) were identified with a threshold of |log2FC| ≥ 1 and FDR < 0.05 (DESeq2/edgeR). Functional enrichment of DEGs was analyzed via Gene Ontology (GO) using clusterProfiler (v4.0).

### Protein extraction and western blotting

For cortical and hippocampal tissues or cells, RIPA lysis buffer was added to extract total protein or histones, according to the manufacturer’s instructions (PK10022, Proteintech). The protein concentration was determined using the BCA method. Proteins were separated by 8% to 15% SDS-PAGE gel electrophoresis (Bio-Rad, Hercules, CA, USA) and transferred to PVDF membranes (Millipore, Boston, MA, USA). The membranes were blocked in 5% BSA for 2 hours at room temperature and incubated with primary antibodies at 4°C overnight. Relative protein expression was normalized to GAPDH (total protein) or H3 (histones). The antibodies used were as follows: rabbit anti-H3 (1:3000, CST, Cat# D1H2), rabbit anti-H3K27ac (1:1000, CST, Cat# D5E4), rabbit anti-GFAP (1:2000, Proteintech, Cat# 16825-1-AP, RRID: AB_2109646)), rabbit anti-Tau-5 (1:1000, Beyotime, Cat# AF1249), rabbit anti-pS199-Tau (1:1000, Beyotime, Cat# AG2590), rabbit anti-pT231-Tau (1:1000, Beyotime, Cat# AF1951), rabbit anti-pS396-Tau (1:1000, Beyotime, Cat# AF2368), rabbit anti-GSK3β (1:1000, CST, Cat# D5C5Z), rabbit anti-pS9-GSK3β (1:1000, Wanleibio, Cat# WL03683, RRID:AB_2928098), rabbit anti-Aβ (anti-Aβ, 1:1000, Thermo Fisher Scientific, Waltham, MA, USA, Cat# 36-6900, RRID: AB_2533275), rabbit anti-amyloid precursor protein (anti-APP, 1:1000, Abways, Shanghai, China, Cat# CY5248), and rabbit anti-GAPDH (1:10,000, Proteintech, Cat# 10494-1-AP, RRID: AB_2263076). The next day, the membrane was washed and incubated with goat anti-rabbit IgG (H+L) (1:10000, Bioss, Cat# bs-80295G-horseradish peroxidase) at room temperature for 1 hour. Immunoreactive bands were detected using an Tanon ECL kit (Tanon, Shanghai, China), images were collected with a ChemiDoc XRS system (Bio-Rad), and band density was quantified using ImageJ software.

### Statistical analyses

The experimental data were statistically analyzed using IBM SPSS Statistics 27 software, and the graphs were drawn using GraphPad Prism 10.1.2 software (GraphPad, San Diego, CA, USA, www.graphpad.com). The data were tested for normality and variance. Independent sample *t* test or Mann–Whitney *U* test was used to compare two groups. One-way analysis of variance with the least significant difference test was used to compare multiple groups. The results are expressed as mean ± SD or median and interquartile range. *P* < 0.05 indicated a statistically significant difference.

## Results

### High-methionine diet consumption induces cognitive impairment in mice

**Figure 1:**
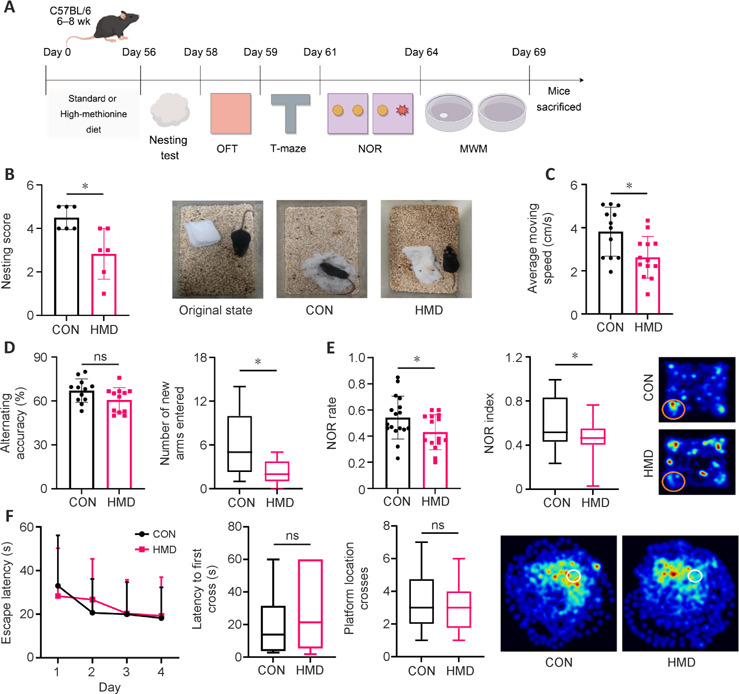
A high-methionine diet induces cognitive impairment in mice. (A) Schematic diagram of the mouse behavior experiment. (B) Nesting test scores and representative images of mice in the HMD and control groups. (C) Average movement speed of the mice in the open field test (OFT) in the HMD and control groups. (D) The spontaneous alternation accuracy rate and the number of new arms entered by the mice in the T maze test in the HMD and control groups. (E) New object recognition (NOR) rate, new object recognition index and group average heatmap in the new object recognition experiment (the circle represents the area where the new object is located) in the HMD and control groups. (F) Escape latency during the training period, latency to first platform crossing and number of platform location crosses during the experimental period, and group average heatmap of the swimming trajectories of the mice in the positioning navigation experiment (the circle represents the platform area). *n* = 6–13. All values are presented as the mean ± SD or median and interquartile range. *P < 0.05 (independent sample *t*-test or Mann‒Whitney *U* test). CON: Control; HMD: high-methionine diet; ns: not significant.

To investigate how Hcy regulates animal behavior, we first established a high-Hcy mouse model by feeding 57BL/6 female mice a high-methionine diet (HMD) for 2 months (**Figure 1A**). Their body weights initially increased but subsequently decreased compared with those of the control mice (**Additional Figure 1A**). The blood glucose concentration was decreased compared with that of control mice beginning on day 14 (**Additional Figure 1A**). Next, we assessed the Hcy concentration in mouse blood and brain tissue to determine whether Hcy was produced via the methionine cycle. Compared with the control group, the HMD group presented increased Hcy levels in both the blood (*P* < 0.0001) and brain (*P* = 0.0002; **Additional Figure 1B**). In addition, there were no significant differences in the brain, heart, liver, and kidney indices between the two groups (**Additional Figure 1C** and **D**). In addition, serum triglyceride and high-density lipoprotein levels were similar in HMD-fed mice and control mice, while total cholesterol and low-density lipoproteinlevels were significantly different between the two groups (*P* = 0.0311 and *P* < 0.0001; **Additional Figure 1E**). This is consistent with an earlier report that elevated serum Hcy concentrations are associated with dyslipidemia (Yuan et al., 2020).

Hcy is a risk factor for cognitive impairment; therefore, we evaluated the cognitive function of HMD mice via behavioral assays (Lan et al., 2022). Compared with the mice in the control group, the autonomic behaviors and social abilities observed in the nesting experiments were significantly lower in the mice fed an HMD for 8 weeks (*P* = 0.0101; **Figure 1B**). The open field test results revealed that hHcy caused decreased voluntary behaviors (*P* = 0.0096; **Figure 1C**). In the T-maze test, compared with the control group, the HMD group performed well in the spontaneous alternation experiment but showed decreased short-term memory in the new arm exploration experiment, as indicated by a decreased number of entries into the new arm (*P* = 0.0157; **Figure 1D**). The novel object recognition and Morris water maze tests showed that, compared with the control group, long-term memory was decreased in the HMD group; in particular, the novel object recognition rate was significantly decreased (*P* = 0.0444; **Figure 1E** and **F**). In summary, these results confirmed that non-spatial memory and long-term memory decreased in mice fed an HMD compared with control mice, indicating cognitive impairment induced by increasing Hcy concentrations.

### Increased homocysteine levels in the brain cause nerve injury and inflammation in high-methionine diet mice

**Figure 2:**
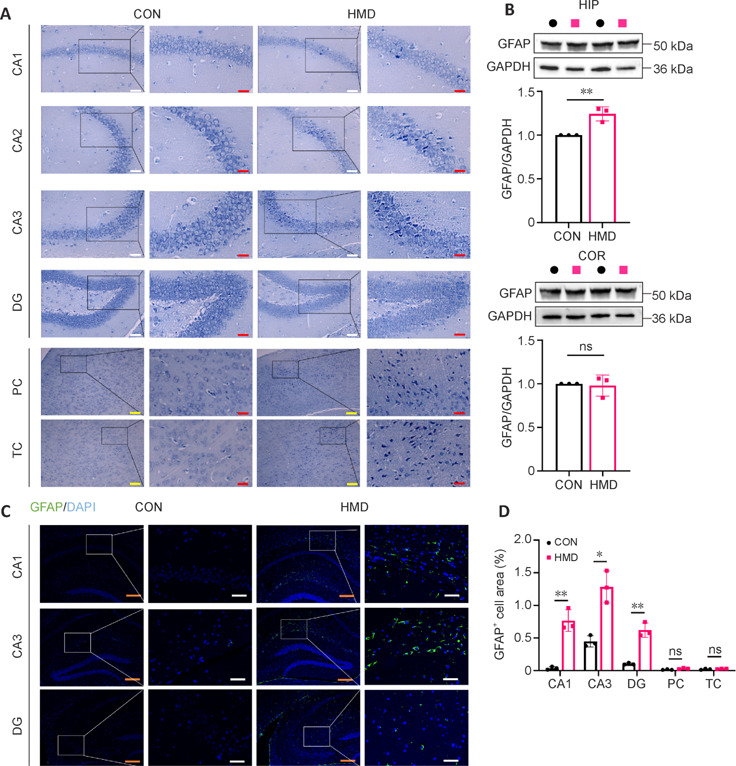
Increased homocysteine levels in the brains of high-methionine diet mice cause nerve injury and inflammation. (A) Representative images of Nissl staining of the CA1, CA2, CA3 and DG regions of the hippocampus, as well as the PC and the TC. Scale bars: red, 25 μm; white, 50 μm; yellow, 100 μm. (B) GFAP expression in the hippocampus and cortex were measured by western blotting (upper panel). Relative protein content of GFAP in the hippocampus and cortex (lower panel). (C) Representative images of GFAP (green) and DAPI (blue) immunostaining in the hippocampi of HMD mice. Scale bars: white, 50 μm; orange, 200 μm. (D) Statistical analysis of the GFAP-positive cell area in the hippocampus and cortex. *n* = 3. All values are presented as the mean ± SD. *P < 0.05, **P < 0.01 (independent sample *t*-test). CON: Control; COR: cortex; DAPI: 4′,6-diamidino-2-phenylindole; DG: dentate gyrus; GFAP: glial fibrillary acidic protein; HIP: hippocampus; ns: not significant; PC: parietal lobe cortex; TC: temporal cortex.

Selective vulnerability of specific neuronal subtypes is a hallmark of NDDs (Beesley et al., 2022). A consistent pattern of selective vulnerability has been observed in the dorsal hippocampus of rodent models of various conditions, such as ischemic damage: neurons in the CA1 region are predominantly affected, while those in the CA3 region and granule cells are spared. The CA3 and dentate gyrus (DG) regions of the hippocampus are involved in spatial learning and memory, which are highly important for maintaining hippocampal function. Therefore, we harvested the brains from mice fed an HMD; divided the hippocampus into the CA1, CA2, CA3, and DG regions and the cortex into the parietal lobe cortex and temporal cortex (**Additional Figure 2A**); and performed Nissl staining and HE staining to detect neuronal degeneration in each of these regions. Both staining approaches revealed obvious nuclear pyknoses, disrupted nuclear membrane structure, and irregular nuclear edges in the HMD group, but not in the control group (**Figure 2A** and **Additional Figure 2B**). We further evaluated levels of GFAP, a protein marker of astrocyte activation, in the hippocampus and cortex (Jurga et al., 2021). Western blotting revealed increased GFAP levels in the hippocampus (*P* = 0.0066) in the HMD group, but not in the cortex, compared with the control group (**Figure 2B**). Immunofluorescence analysis confirmed the western blotting results. In addition, more GFAP-positive cells were observed in the hippocampal region of HMD mice than in that of control mice, and astrocytes were scattered throughout the hippocampal region, especially in CA1, CA3, and the DG; however, there were no obvious differences in the parietal lobe cortex or temporal cortex between the two groups (**Figure** **2C** and **D** and **Additional Figure 2C**). These results suggest that astrocyte activation and neuroinflammation occur in the hippocampus in mice fed an HMD, which may represent a protective response against increased Hcy levels in brain tissue.

### Increased Tau protein deposition in the hippocampi and cortices of high-methionine diet mice

**Figure 3:**
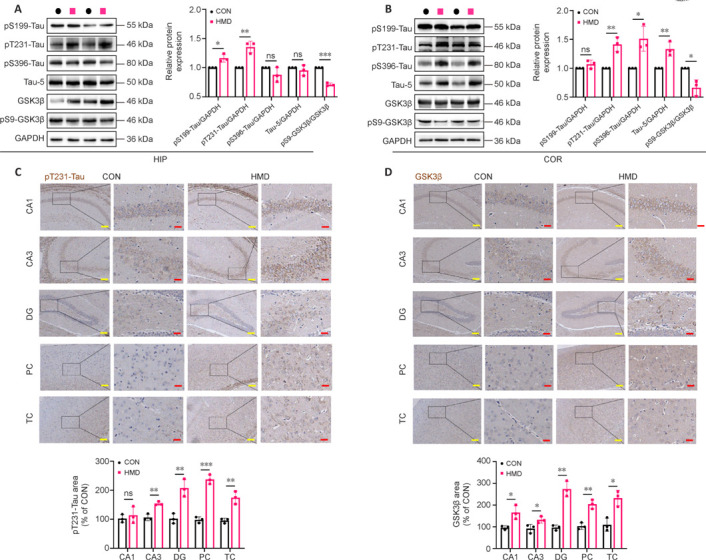
Increased Tau protein deposition in the hippocampus and cortex of high-methionine diet mice. (A, B) pS199-Tau, pT231-Tau, pS396-Tau, Tau-5, GSK-3β and pS9-GSK-3β protein expression in the hippocampus and cortex were measured via western blotting (left panel). Relative protein expression of pS199-Tau, pT231-Tau, pS396-Tau, Tau-5, GSK-3β and pS9-GSK-3β in the hippocampus and cortex (right panel). (C, D) Representative immunohistochemical images (upper panel) and their relative positive expression (lower panel) of pT231-Tau and GSK-3β in the hippocampus and cortex. Scale bars: red, 25 μm; yellow, 100 μm. *n* = 3. All values are presented as the mean ± SD. *P < 0.05, **P < 0.01, ***P < 0.001 (independent sample *t*-test). CON: Control; COR: cortex; GSK-3β: glycogen synthase kinase-3β; HIP: hippocampus; HMD: high-methionine diet; ns: not significant; pS199-Tau: phospho-Tau (Serine 199); pS396-Tau: phospho-Tau (Serine 396); pS9-GSK-3β: phospho-GSK-3β (Serine 9); pT231-Tau: phospho-Tau (Threonine 231); Tau-5: Pan-Tau.

To determine whether HMD-induced cognitive impairment is caused by the formation of neurotoxic Tau protein aggregates (Dang et al., 2023), we first evaluated Tau protein levels in the hippocampus via western blotting. The total Tau protein (Tau-5) content was not significantly different between the HMD and control groups (**Figure 3A**). Subsequently, we performed western blotting for Tau protein phosphorylated at S199, T231, or S396 (Weidling et al., 2020; Fu et al., 2023; Wang et al., 2023), all of which indicate cognitive dysfunction. pS199-Tau (*P* = 0.0142) and pT231-Tau (*P* = 0.0034) protein levels were increased in HMD mice compared with control mice (**Figure 3A**), suggesting cognitive impairment, but pS396-Tau levels were not. Similar results were observed in the cortex: total Tau protein (*P* = 0.0095), pT231-Tau (*P* = 0.0047), and pS396-Tau (*P* = 0.0152) levels were significantly increased, whereas pS199-Tau levels were not significantly different, in the HMD group compared with the control group (**Figure 3B**). Abnormal GSK-3β activation has been reported to lead to increased Aβ production, Tau hyperphosphorylation, and induction of apoptosis and inflammation, via multiple signaling pathways (Gandy et al., 2013). Therefore, we also performed western blotting for GSK-3β and its phosphorylated counterpart (pS9-GSK-3β). In both the hippocampus and cortex of HMD mice, the ratio of pS9-GSK-3β to GSK-3β was significantly decreased compared to the hippocampus and cortex from control mice (*P* < 0.0001, *P* = 0.0128; **Figure 3A** and **B**). To verify these changes, we assessed pT231-Tau and GSK-3β levels in different hippocampal and cortical regions via IHC staining and found that the levels of both proteins were increased in the HMD mice hippocampus and cortex (**Figure 3C** and **D**). However, APP and Aβ protein levels did not differ between the hippocampus and cortex (**Additional Figure 3A** and **B**). Taken together, these results indicate that, in HMD mice, cognitive impairment may result from increased Tau protein and decreased phosphorylated GSK-3β levels.

### Metabolomic alterations in the hippocampi of high-methionine diet mice coordinated with the methionine cycle

**Figure 4:**
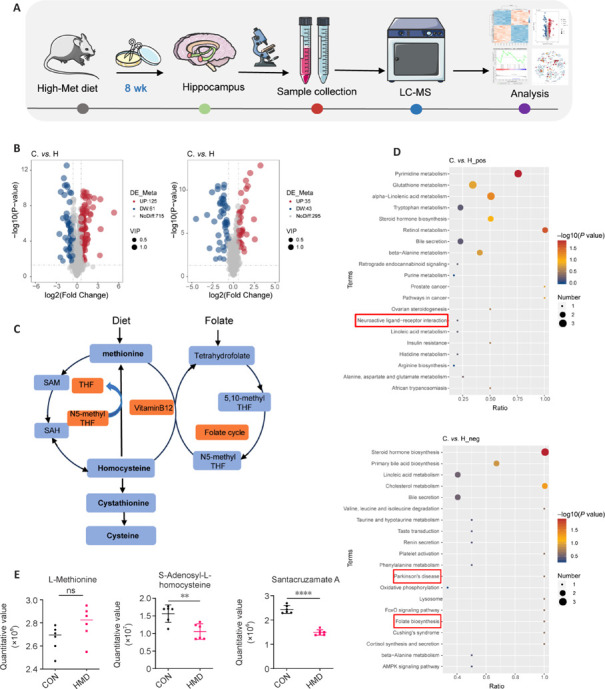
Metabolomic alterations in the hippocampus of high-methionine (Met) diet mice. (A) Schematic diagram of the metabolomics sequencing strategy for the mouse hippocampus. (B) Volcano plots showing that among 901 and 373 metabolites identified in positive-ion mode (left) and negative-ion (right) mode, respectively, differentially abundant metabolites were screened out according to the criteria of VIP > 1.0, FC > 1.5 or FC < 0.667 and *P*-value < 0.05. The red dots represent upregulated metabolites, and the blue dots represent downregulated metabolites compared with those in the HMD group. (C) Schematic diagram of the methionine cycle. (D) KEGG pathway enrichment analysis of differentially abundant metabolite was used to identify the top 20 differential pathways between the two groups in positive and negative ion modes. (E) Quantification of three metabolites, SAH, L-methionine, and santacruzamate A in the control and HMD groups. *n* = 6. All values are presented as the mean ± SD. **P < 0.01, ****P < 0.0001 (independent sample *t*-test). CON: Control; FC: fold change; HMD: high-methionine diet; KEGG: Kyoto Encyclopedia of Genes and Genomes; ns: not significant; SAH: L-methionine and S-adenosyl-L-homocysteine; VIP: variable importance in projection.

In recent years, metabolism-driven epigenomic events have been shown to regulate NDD occurrence and development (Li et al., 2020a; Baloni et al., 2021). To determine whether Hcy regulates metabolism in the mouse hippocampus, we performed LC‒MS (**Figure 4A**) and identified 186 and 78 differentially abundant metabolites between the HMD group and the control group in positive-ion mode and negative-ion mode, respectively (**Figure 4B**). To elucidate how these metabolites regulate cognitive impairment, we performed Kyoto Encyclopedia of Genes and Genomes (KEGG) pathway enrichment analysis. The differentially abundant metabolites detected in positive-ion mode were mainly enriched in pyrimidine metabolism, glutathione metabolism, neuroactive ligand‒receptor interaction, and other metabolic pathways, indicating that the methionine cycle and related metabolites in the hippocampus were influenced by HMD consumption. Unexpectedly, the metabolites detected in negative-ion mode were enriched in the PD and folate biosynthesis pathways (**Figure 4D**). Glutathione metabolism and folate biosynthesis are involved in the methionine cycle (**Figure 4C**). These analyses suggest that HMD consumption induces the production of metabolites via the methionine cycle that contribute to cognitive impairment.

Subsequently, we quantified the levels of metabolites related to the methionine cycle and found that L-methionine was increased in the HMD group, although the difference was not significant, whereas S-adenosyl-homocysteine (SAH) were significantly decreased in the HMD group compared with the control group (*P* = 0.0040; **Figure 4E**). L-methionine reportedly enhances neuroinflammation and impairs neurogenesis (Alachkar et al., 2022), and our results confirmed this conclusion. In addition, decreased S-adenosyl-methionine and increased SAH levels can be caused by high Hcy and folate or vitamin B12 deficiency, which inhibits DNA methylation and leads to hippocampal neurogenesis (Gou et al., 2021). We found that the level of santacruzamate A, an HDAC inhibitor that exerts neuroprotective effects by targeting Aβ (Pavlik et al., 2013), was significantly lower in the HMD group than in the control group (*P* < 0.0001; **Figure 4E**). These findings indicate that HMD consumption decreases innate neuroprotection and acetylation in the context of cognitive impairment and NDDs. In summary, our findings show that HMD-induced methionine cycle dysfunction contributes to cognitive impairment.

### High homocysteine induces cognitive impairment by downregulating H3K27ac and genes involved in synaptic long-term potentiation

**Figure 5:**
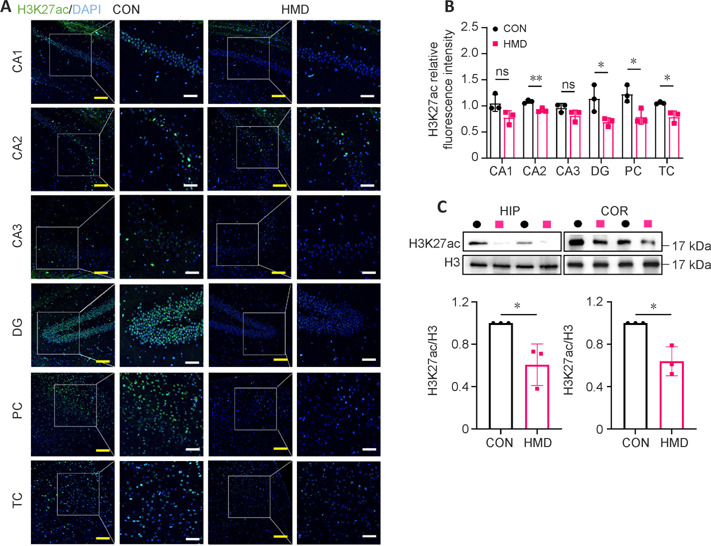
High homocysteine levels induce cognitive impairment in mouse brain tissue by downregulating H3K27ac. (A) Representative images of H3K27ac (green) and DAPI (blue) immunofluorescence staining in the hippocampus and cortex. Scale bars: white, 50 μm; yellow, 100 μm. (B) Statistical analysis of the immunofluorescence staining intensity of H3K27ac. (C) Relative protein expression of H3K27ac in the hippocampus and cortex groups. *n* = 3. All values are presented as the mean ± SD. *P < 0.05, **P < 0.01 (independent sample *t*-test). CON: Control; COR: cortex; DAPI: 4′,6-diamidino-2-phenylindole; HIP: hippocampus; HMD: high-methionine diet; ns: not significant.

The HDAC inhibitor santacruzamate A was downregulated in HMD mice, and histone acetylation is essential for maintaining neuronal plasticity and memory (Kabir et al., 2022). Therefore, we investigated H3K27ac, a PTM correlated with NDDs (Cheng et al., 2018), in the hippocampus of HMD mice via immunofluorescence and western blotting. Immunofluorescence analysis showed that H3K27ac was decreased in four regions of the hippocampus and two regions of the cortex (**Figure 5A**). This was confirmed by a decreased in estimated H3K27ac relative fluorescence intensity in these six regions (**Figure 5B**). Additionally, western blot analysis of hippocampal and cortical tissues revealed significantly lower H3K27ac protein levels in HMD mice than in control mice (*P* = 0.0257, *P* = 0.0105; **Figure 5C**). Taken together, these results suggest that low H3K27ac protein levels may result in cognitive impairment in HMD mice.

**Figure 6:**
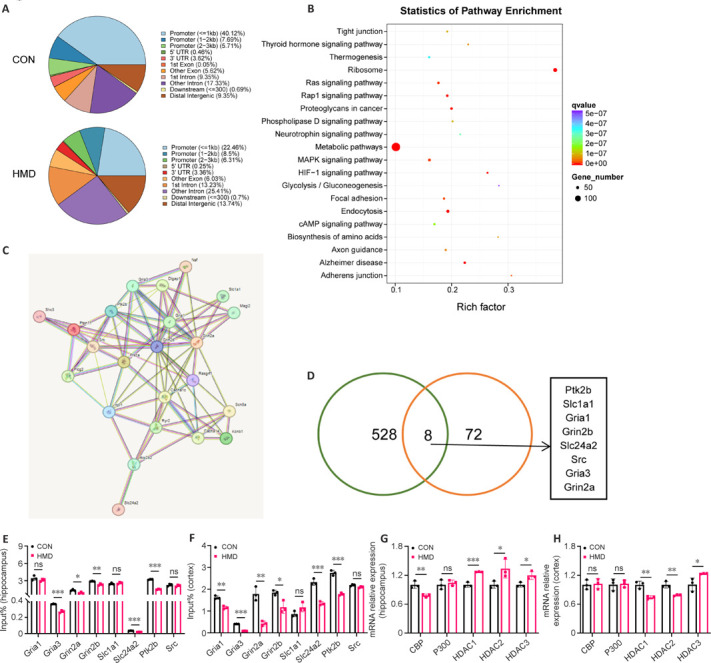
Downregulation of H3K27ac targets genes involved in synaptic long-term potentiation in the brain of high-methionine diet mice. (A) Pie charts of H3K27ac distribution in CON and HMD mice. (B) KEGG analysis of genes with different H3K27ac peaks in the hippocampal tissues of CON and HMD mice. (C) STRING analysis of genes with different H3K27ac peaks revealed a protein interaction network of gene products. (D) Venn diagram showing the overlapping target genes between genes with significantly decreased H3K27ac peaks and synaptic long-term potentiation genes (GO:0060291), including *Gria1*, *Gria3*, *Grin2a*, *Grin2b*, *Slc1a1*, *Slc24a2*, *Ptk2b*, and *Src*. (E, F) ChIP-qPCR quantification of H3K27ac enrichment in enhancer regions of overlapping target genes in the hippocampus and cortex from control and HMD mice. (G, H) qRT-PCR analysis of enzymes associated with acetylation gene expression in the hippocampus and cortex from control and HMD mice. GAPDH was used as a loading control. *n* = 3. All values are presented as the mean ± SD. *P < 0.05, **P < 0.01 (independent sample *t*-test). CBP: CREB-binding protein; ChIP-qPCR: chromatin immunoprecipitation-qPCR; CON: control; GAPDH: glyceraldehyde 3-phosphate dehydrogenase; H3K27ac: histone H3 lysine 27 acetylation; HDAC: histone deacetylase; HMD: high-methionine diet; KEGG: Kyoto Encyclopedia of Genes and Genomes; ns: not significant.

Decreased H3K27ac, a marker of active epigenetic enhancers, decreases gene transcription (Zhang et al., 2020). To further evaluate the decrease in H3K27ac function and identify its target genes in HMD mice, we performed H3K27ac ChIP-seq of hippocampal tissue. The distribution of H3K27ac peaks in the distal intergenic region was different in the control group and the HMD group (**Figure 6A**), suggesting that H3K27ac is marker of enhanced transcription. In addition, pathway enrichment analysis of the different H3K27ac peaks revealed that the neurotrophins and AD signaling pathways were among the top 20 enriched pathways (**Figure 6B**). We also screened for the most significantly decreased H3K27ac peak genes via the STRING database and found that the peaks were enriched in genes involved in synaptic long-term potentiation (**Figure 6C**). Venn diagram analysis of genes with decreased H3K27ac peaks and genes involved in synaptic long-term potentiation identified eight common genes, namely *Ptk2b*, *Slc1a1*, *Gria1*, *Gri2b*, *Slc24a2*, *Src*, *Gria3*, and *Grin2a* (**Figure 6D**). To investigate whether transcription of these genes was induced by decreased H3K27ac in their enhancer regions, we designed specific primers for these eight genes that targeted the decreased H3K27ac peaks via ChIP-seq. We then performed ChIP‒qPCR with an anti-H3K27ac antibody on hippocampal and cortical tissues and confirmed that H3K27ac enrichment in the enhancer regions of *Gria3* (*P* = 0.0005, *P* < 0.0001), *Grin2a* (*P* = 0.0194, *P* = 0.0023), *Grin2b* (*P* = 0.0035, *P* = 0.0187), *Slc24a2* (*P* = 0.0009, *P* = 0.0005), and *Ptk2b* (*P* < 0.0001, *P* = 0.0002) was significantly lower in the HMD group than in the control group (**Figure 6E** and **F**). Finally, we performed qRT-PCR and found that HDAC3 may be responsible for decreased H3K27ac enrichment in these regions (**Figure 6G** and **H**). Collectively, these results suggest decreased H3K27ac binding to genes involved in synaptic long-term potentiation, contributing to cognitive impairment.

**Figure 7:**
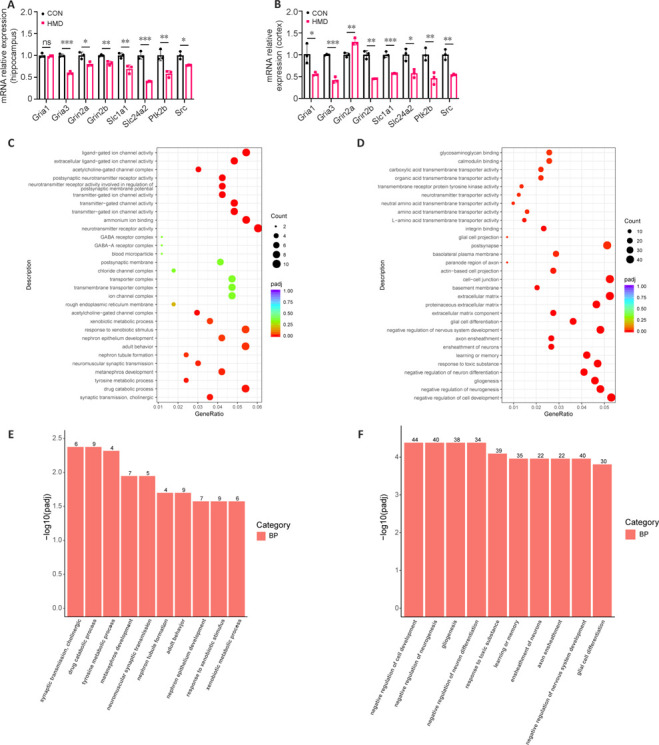
High-methionine diet induces pathological neurodegeneration in the hippocampus and cortex of mice. (A, B) Quantitative reverse transcription-polymerase chain reaction analysis of genes involved in long-term synaptic potentiation in the hippocampus and cortex in control and HMD mice. Glyceraldehyde 3-phosphate dehydrogenase was used as a loading control. *n* = 3. (C, D) KEGG analysis of differentially expressed genes in the hippocampi and cortices of HMD mice using RNA-seq. (E, F) GO analysis of differentially expressed genes in the hippocampus and cortex of HMD mice using RNA-seq. BP represents a biological process term in the GO analysis. All values are presented as the mean ± SD. *P < 0.05, **P < 0.01, ***P < 0.001 (independent sample *t*-test). GAPDH was used as a loading control. *n* = 3. CON: Control; GAPDH: glyceraldehyde 3-phosphate dehydrogenase; GO: Gene Ontology; HMD: high-methionine diet; KEGG: Kyoto Encyclopedia of Genes and Genomes; ns: not significant.

To confirm the regulatory effect of H3K27ac on cognitive impairment, we first performed RT-qPCR to evaluate transcription of genes involved in synaptic long-term potentiation. As shown in **Figure 7A** and **B**, most of the eight genes exhibited decreased transcription in the hippocampus and cortex. RNA-seq analysis of the hippocampus and cortex of HMD mice showed that these genes were enriched in different pathways in the hippocampus, including ligand-gated channel activity, neurotransmitter receptor activity, the GABA receptor complex, and neuromuscular synaptic transmission (**Figure 7C**). Similar results were also observed in the cortex, with the enriched pathways including learning from memory, axon establishment, and negative regulation of neuron differentiation (**Figure 7D**). Finally, Gene Ontology (GO) analysis of biological processes in the hippocampus and cortex confirmed these results (**Figure 7E** and **F**), indicating that hHcy driven by consumption of an HMD leads to cognitive impairment–related pathological processes.

### High homocysteine promotes neurotoxicity in HT-22 cells

**Figure 8:**
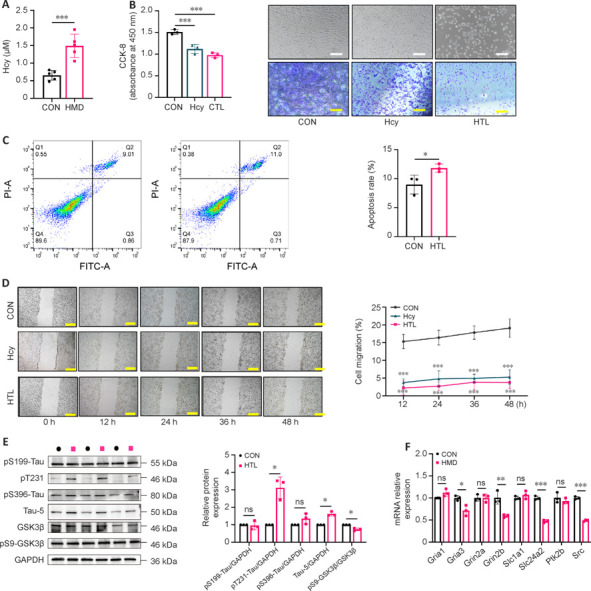
High-methionine diet mediates neurotoxicity by inhibiting proliferation and promoting apoptosis in HT-22 cells. (A) Hcy concentrations in cells after 2 mM HTL treatment, as determined via enzyme-linked immunosorbent assay. (B) CCK-8 results and representative images of crystal violet staining after 2 mM Hcy or HTL treatment in HT-22 cells. Scale bars: 100 μm. (C) HT-22 apoptosis was examined via flow cytometry after 2 mM HTL treatment (left panel). The percentage of apoptotic cells was quantified (right panel). (D) Representative images of cell migration by the cell scratch assay (left panel) and quantification of cell migration with 2 mM Hcy or HTL treatment. (E) Protein expression of pS199-Tau, pT231-Tau, pS396-Tau, Tau-5, GSK-3β, pS9-GSK-3β, and GAPDH in HT-22 cells treated with 2 mM HTL were measured via western blotting (left panel). Quantification of protein expression in HT-22 cells treated with 2 mM HTL (right panel). (F) Quantitative reverse transcription-polymerase chain reaction analysis of the expression of genes involved in long-term synaptic potentiation in the control and high-methionine diet groups. GAPDH was used as a loading control, *n* = 3. All values are presented as the mean ± SD. *P < 0.05, **P < 0.01, ***P < 0.001 (one-way analysis of variance with the least significant difference test). CCK-8: Cell Counting Kit-8; CON: control; GAPDH: glyceraldehyde 3-phosphate dehydrogenase; Hcy: homocysteine; HMD: high-methionine diet; HTL: homocysteine thiolactone; ns: not significant.

Next, we performed *in vitro* experiments to confirm our *in vivo* findings. We chose HTL, an intermediate metabolite in the Hcy metabolic pathway that has been reported to react with the ε-amino group of the lysine residue in proteins, to represent hHcy. In accordance with previous studies (Chai et al., 2024; Yang et al., 2024), we first treated HT-22 cells with 0.1, 0.5, 1, or 2 mM HTL or Hcy. CCK-8 cell viability assay showed that proliferation was significantly decreased in HT-22 cells treated with 2 mM HTL or Hcy (**Additional Figure 4**). ELISA also revealed a significant increase in Hcy concentrations in HT-22 cells treated with 2 mM HTL compared with the control group (*P* < 0.001; **Figure 8A**). To investigate hHcy-induced toxicity in HT-22 cells, cell proliferation and apoptosis were evaluated. Both CCK-8 and crystal violet staining were significantly decreased (**Figure 8B**), indicating that treatment with hHcy decreased proliferation. Similarly, scratch assay and apoptosis experiments revealed that cell migration declined over time in cells treated with 2 mM hHcy (**Figure 8D**), and that apoptosis also increased in hHcy-treated cells (**Figure 8C**). These results suggest that treatment with 2 mM HTL or Hcy was toxic to HT-22 cells, contributing to decreased proliferation and increased apoptosis.

Next, we evaluated pS199-Tau, pT231-Tau, and pS396-Tau protein levels in HT-22 cells treated with hHcy. Western blotting showed that pT231-Tau (*P* = 0.027) and Tau-5 (*P* = 0.012) protein levels increased, whereas no changes in pS199-Tau or pS396-Tau protein levels were detected (**Figure 8E**). Furthermore, western blot analysis showed that the ratio of pS9-GSK-3β to GSK-3β was significantly decreased in hHcy-treated HT-22 cells, which is in accordance with the results from the hippocampus and cortex (*P* = 0.0351; **Figure 8E**). Finally, the mRNA levels of genes involved in synaptic long-term potentiation were assessed by qRT-PCR, and the results revealed that the levels of *Gria3* (*P* = 0.0203), *Grin2b* (*P* = 0.0089), *Slc24a2* (*P* < 0.0001), and *Src* (*P* < 0.0001) decreased significantly (**Figure 8F**). Collectively, these *in vitro* results suggest that hHcy promotes neurotoxicity.

## Discussion

The etiology and pathogenesis of cognitive impairment remain unclear, which hinders progress in developing new treatments and improving currently available treatments. Over the past decade, several reports have demonstrated the key roles of Hcy in cellular function and the molecular mechanisms of cognitive impairment and NDD (Lin et al., 2022; Li et al., 2023; Perinan et al., 2023). Here, we used a mouse model of HMD-induced cognitive impairment to investigate the role of the epigenetic histone modification H3K27ac, which regulates N-methyl-D-aspartate receptor expression and function, thereby mediating cognitive impairment (Li et al., 2020b). Our study yielded several interesting and novel findings: i. HMD mice exhibited nerve injury and inflammation; ii. Tau phosphorylation was involved in HMD-induced cognitive impairment in a mouse model; iii. Mice with Hcy-induced cognitive impairment exhibited alterations in methionine cycle–related metabolite and HDAC inhibitor levels; and iv. decreasing H3K27ac binding to genes involved in synaptic long-term potentiation with HDAC inhibitors contributed to HMD-induced cognitive impairment. In summary, our findings show that crosstalk between metabolomics and epigenetics mediates Hcy-induced cognitive impairment. These findings improve our understanding of the role of Hcy in neurodegeneration, potentially providing new insights into the clinical diagnosis and treatment of this condition.

Cognitive decline and neuropathological protein accumulation are the main pathological processes contributing to NDD (Shao et al., 2025; Tinu et al., 2025). Gonzales et al. (2022) reported that sustained neuroinflammation and nerve injury are correlated with susceptibility to NDD. Here, we evaluated the expression of GFAP, an astrocyte marker, via western blotting and immunofluorescence and found that GFAP expression was significantly increased in the hippocampus but not in the cortex. Nissl staining and IHC analysis revealed obvious nuclear pyroptosis, disrupted nuclear membrane structure, and irregular edges in the HMD-induced group. These results suggest that astrocyte activation and neuroinflammation occurred in the hippocampi of HMD mice, which may be a protective response to changes in Hcy levels in brain tissue. This finding was consistent with astrocytes having neuroprotective, angiogenic, immunomodulatory, neurogenic, and antioxidant properties and modulate synaptic function when activated (Becerra-Calixto and Cardona-Gomez, 2017).

Tau protein forms pathological fibrillar aggregates that are strongly linked to neuronal cell death and contribute to diverse clinical phenotypes, such as dementia, movement disorders, and motor neuron disease (Creekmore et al., 2024). Tau protein is subject to numerous PTMs, such as hyperphosphorylation, which leads to the formation of paired helical filaments and neurofibrillary tangles and contributes to cognitive impairment (Wang and Mandelkow, 2016; Stefanoska et al., 2022). In addition, phosphorylation of Tau protein can drive activation of GSK-3β, a key player in signaling pathways required for normal brain function and a critical molecular link between Aβ and neurofibrillary tangles (Chauhan et al., 2022). GSK-3β inhibits Ser9 phosphorylation (Krishnankutty et al., 2017). Therefore, to characterize the detailed mechanism underlying HMD-induced cognitive impairment, we evaluated pS199-Tau, pT231-Tau, pS396-Tau, total Tau protein, Aβ, total GSK-3β, and pS9-GSK-3β levels in the hippocampus and cortex. The results suggested that increased Tau protein and activated GSK-3β levels may be strongly correlated with cognitive impairment in HMD mice, but that Aβ is not involved. These results confirm that abnormal protein aggregation and activation contribute to impaired hippocampal and cortical function in the contest of cognitive impairment.

Neuronal function and differentiation are tightly regulated by both the metabolome and the epigenome (Ghosh and Saadat, 2023). Changes in nutrient intake lead to metabolomic and epigenetic alterations. Therefore, neurodegeneration is the consequence of both genomic and epigenomic dysregulation. Mice with hHcy induced by consumption of a Met-enriched diet for 4 weeks exhibited altered histomorphology and metabolic profiles in the hippocampus and decreased neuronal vitality, which could eventually lead to neurodegeneration (Kovalska et al., 2019, 2021). We found that differentially abundant metabolites induced by hHcy were enriched in neuroactive ligand‒receptor interactions and PD. Interestingly, the differentially abundant metabolites were also enriched in folate biosynthesis, which confirmed that hHcy disrupted the methionine cycle. Furthermore, L-methionine levels were increased in the hHcy group, whereas SAH levels were significantly decreased. The decreased SAH levels may be attributed to folate deficiency and not by hHcy, which was is induced by consumption of a Met-enriched diet for 8 weeks.

Metabolomic analysis of the mice that consumed a Met-enriched diet showed that reduced glucose utilization is compensated for by increased triacylglycerol metabolism. This metabolic shift coordinates cellular function through epigenomic regulation (Ruan and Crawford, 2018), particularly by influencing histone acetylation (Su et al., 2016), thereby modulating the growth, apoptosis, and survival of glial cells and neurons (Yang et al., 2019). We found that the level of santacruzamate A, an HDAC inhibitor, was significantly decreased. A recent study has shown that Tau protein affects histone acetylation and chromatin structure in the prefrontal cortex of patients with AD (Klein et al., 2019). Differential H3K27ac peaks were also associated with AD-related genes, which implies that H3K27ac is involved in neuropathology (Marzi et al., 2018). On the basis of these findings and our metabolomic results, we performed H3K27ac-ChIP-seq and found decreased H3K27ac binding to, and therefore decreased transcription of, genes involved in synaptic long-term potentiation. Additionally, the top 20 pathways enriched in downregulated H3K27ac peaks included AD and axon guidance, which is consistent with findings reported by Marzi et al. (2018). These results suggest that high homocysteine drives epigenomic and metabolomic changes in the hippocampus that contribute to abnormal gene expression and lead to cognitive impairment.

Our study has several limitations that should be considered when interpreting the results. First, we did not have enough brain samples from patients with clinical cognitive impairment to confirm our results because most patients with cognitive impairment are medicated. Second, metabolomics and ChIP-seq analyses were performed on whole hippocampal, and specific areas, such as CA1, CA2, CA3, or DG, should be analyzed to reveal their specific functions. In addition, further investigations of folate rescue or other neuroprotective factors, such as physical activity, should be performed to elucidate the associations between decreased H3K27ac and differentially abundant metabolites. Finally, the 2 mM Hcy used *in vitro* exceeds the levels of Hcy detected in the brains of HMD mice (50.49 ± 14.23 μM), which is likely due to *in vivo* protective mechanisms. While the concentration used *in vitro* enabled us to obtain important mechanistic insights, chronic exposure to lower, physiological levels may still cause cumulative neuronal damage. Future studies should explore the prolonged effects of Hcy at physiologically relevant concentrations to better bridge *in vitro* and *in vivo* findings.

In summary, our study focused on the effects of HMD-induced hHcy on behavioral changes and the underlying mechanisms involved. Our data suggest that changes in the transcription of genes involved in synaptic long-term potentiation driven by H3K27ac contribute to cognitive impairment, imply that H3K27ac could be a useful drug target. In addition to identifying the molecular mechanisms associated with neuropathology in cognitive impairment, we present a framework for metabolomic–epigenetic–transcription studies of complex diseases, suggesting potential targets for clinical treatment and highlighting potential avenues for treating cognitive impairment.

## Additional files:

***Additional Figure 1:*** *Increased homocysteine levels in mouse brains induced by a high-methionine diet.*

Additional Figure 1 Increased homocysteine levels in mouse brains induced by a high-methionine diet.
(A) Body weights and random blood glucose levels of CON and HMD mice. (B) Hcy levels in the serum and brain tissue of CON and HMD mice were measured using enzyme-linked immunosorbent assay. (C, D) Brain, heart, liver, and kidney indices of CON and HMD mice. (E) Serum TC, TG, LDL and HDL concentrations of CON and HMD mice, *n*=6-23. All values are presented as the mean ± SD. *P < 0.05, **P < 0.01, ***P < 0.001. CON: Control; HDL: high-density lipoprotein; HMD: high-methionine diet; LDL: low-density lipoprotein; ns: not significant; TC: total cholesterol; TG: triglyceride.

***Additional Figure 2:*** *Increased homocysteine levels in the mouse brain cause nerve injury and inflammation.*

Additional Figure 2 Increased homocysteine levels in the mouse brain cause nerve injury and inflammation.
(A) Schematic diagram of coronal sections of the mouse hippocampus and cortex. The hippocampus is divided into 4 regions, namely, the CA1, CA2, CA3 and DG regions; the cortex is divided into the PC and the TC. (B) Representative images of HE staining of the CA1, CA2, CA3 and DG regions of the hippocampus, as well as the PC and the TC, under a light microscope. Scale bars: red, 25 μm; white, 50 μm; yellow, 100 μm. (C) Representative images of GFAP (green) and DAPI (blue) immunostaining in the cortex. Scale bars: white, 50 μm; orange, 200 μm. CON: Control; DG: dentate gyrus; GFAP: glial fibrillary acidic protein; HMD: high-methionine diet; PC: parietal lobe cortex; TC: temporal cortex.

***Additional Figure 3:*** *Unchanged amyloid-β deposition in the hippocampus and cortex induced by HMD.*

Additional Figure 3 Unchanged amyloid-β deposition in the hippocampus and cortex induced by HMD.
(A, B) Protein expression of APP and amyloid-β in the HMD hippocampus (left) and cortex (right) were determined by western blotting. *n*=3. All values are presented as the mean ± SD. APP: Amyloid precursor protein; CON: control; COR: cortex; HIP: hippocampus; HMD: high-methionine diet; ns: not significant.

***Additional Figure 4:*** *High concentrations of Hcy or HTL induce apoptosis.*

Additional Figure 4 High concentrations of Hcy or HTL induce apoptosis.
Cell viability by Cell Counting Kit-8 (CCK-8) after treatment with 100 μM, 500 μM, 1 mM and 2 mM Hcy or HTL. *n*=3. All values are presented as the means ± SDs. ^**^P<0.01, ^***^P<0.001. CON: Control; Hcy: homocysteine; HTL: homocysteine thiolactone.

***Additional Table 1:*** *Primers used for chromatin immunoprecipitation-quantitative polymerase chain reaction*

***Additional Table 2:*** *Primers used for quantitative reverse transcription-polymerase chain reaction.*

## Data Availability

*All relevant data are within the paper and its Additional files.*
